# Differentially co-expressed interacting protein pairs discriminate samples under distinct stages of HIV type 1 infection

**DOI:** 10.1186/1752-0509-5-S2-S1

**Published:** 2011-12-14

**Authors:** Dukyong Yoon, Hyosil Kim, Haeyoung Suh-Kim, Rae Woong Park, KiYoung Lee

**Affiliations:** 1 Department of Biomedical Informatics, Ajou University School of Medicine, Suwon 443-749, Korea; 2 Department of Anatomy, Ajou University School of Medicine, Suwon 443-749, Korea; 3 Departments of Medicine and Bioengineering, University of California San Diego, La Jolla, California 92093, USA

## Abstract

**Background:**

Microarray analyses based on differentially expressed genes (DEGs) have been widely used to distinguish samples across different cellular conditions. However, studies based on DEGs have not been able to clearly determine significant differences between samples of pathophysiologically similar HIV-1 stages, e.g., between acute and chronic progressive (or AIDS) or between uninfected and clinically latent stages. We here suggest a novel approach to allow such discrimination based on stage-specific genetic features of HIV-1 infection. Our approach is based on co-expression changes of genes known to interact. The method can identify a genetic signature for a single sample as contrasted with existing protein-protein-based analyses with correlational designs.

**Methods:**

Our approach distinguishes each sample using differentially co-expressed interacting protein pairs (DEPs) based on co-expression scores of individual interacting pairs within a sample. The co-expression score has positive value if two genes in a sample are simultaneously up-regulated or down-regulated. And the score has higher absolute value if expression-changing ratios are similar between the two genes. We compared characteristics of DEPs with that of DEGs by evaluating their usefulness in separation of HIV-1 stage. And we identified DEP-based network-modules and their gene-ontology enrichment to find out the HIV-1 stage-specific gene signature.

**Results:**

Based on the DEP approach, we observed clear separation among samples from distinct HIV-1 stages using clustering and principal component analyses. Moreover, the discrimination power of DEPs on the samples (70–100% accuracy) was much higher than that of DEGs (35–45%) using several well-known classifiers. DEP-based network analysis also revealed the HIV-1 stage-specific network modules; the main biological processes were related to “translation,” “RNA splicing,” “mRNA, RNA, and nucleic acid transport,” and “DNA metabolism.” Through the HIV-1 stage-related modules, changing stage-specific patterns of protein interactions could be observed.

**Conclusions:**

DEP-based method discriminated the HIV-1 infection stages clearly, and revealed a HIV-1 stage-specific gene signature. The proposed DEP-based method might complement existing DEG-based approaches in various microarray expression analyses.

## Background

Human immunodeficiency virus type 1 (HIV-1) has been demonstrated to damage the human immune system, finally leading to acquired immunodeficiency syndrome (AIDS), which is characterized by vulnerability to life-threatening opportunistic infections. The natural progression of HIV-1 consists of the acute stage, the clinical latency stage, and AIDS [1]. The acute stage (*Acute*), the first stage of HIV-1 infection, results from contamination with the HIV-1 virus through body fluids such as blood, semen, or vaginal fluid. In this stage, the copy number of HIV-1 virus rapidly increases, and the number of CD4+ T cells markedly decreases [2]. However, most patients with HIV-1 infection recover from the acute stage without treatment within 3 to 6 weeks and have a period of clinical latency of 8 to 10 years [1]. Although there are no clinical manifestations and the CD4+ T-cell count is almost recovered during the clinical latency stage, it has been reported that immune damage persistently occurs [3]. Among the HIV-infected population, approximately 5 to 8% of patients remain clinically stable for decades. They have been referred to as long-term non-progressors (*Non-progressive*) [4]. However, most patients undergo chronic progressive infection (*Chronic*) that finally leads to AIDS, at which point the CD4+ T-cell count drops below 200 cells/μL, and T cell-mediated immunity fails to protect the body from pathogens.

Several studies have attempted to reveal the mechanism of HIV-1 pathogenesis at the genomic level using microarray experiments. Using analysis of differentially expressed genes (DEGs) across HIV-1 infection stages, Hyrcza *et al*. found that expression of interferon-stimulated genes is increased in the early and chronic progressive stages [5]. Li *et al*., by a similar DEG-based analysis using lymphatic tissue microarrays, showed that each stage has relatively different gene expression patterns [6]. These studies have enhanced our knowledge about the pathogenic mechanism of HIV-1. One of the common limits of these studies, however, is that DEG-based expression analysis cannot identify an HIV-1 stage-specific gene signature that can clearly discriminate pathophysiologically similar stages, such as between *Acute* and *Chronic* stages or between *Uninfected* and *Non-progressive* stages [5,6].

Recently, protein-interaction-based analyses with correlational designs have been successfully applied to discover a discriminant genetic signature for a specific condition, but not for an individual sample, using microarray analysis [7,8]. These analyses usually have different assigned weights for an interacting protein pair based on degrees of correlation of expression levels under specific conditions. Genes or gene products do not work alone, but rather function in relationship with other genes or proteins in a real molecular setting [9]. Moreover, the degree of correlation between members of an interacting protein pair under a specific condition might provide evidence for the degree of functional relationship under that condition. However, this approach requires multiple samples under a target condition to extract the genetic features for the condition; thus, it cannot be used for a genetic signature of a single sample, which is required to validate or test whether a new sample has a signature similar to those of other samples in a certain group.

Here, we suggest a novel protein-interaction-based method to capture a genetic signature for a single sample under a specific condition. To achieve this purpose, we assigned a co-expression (or co-changing) score to a protein–protein interaction by comparing the expression-change ratios of the two genes in a sample with representative values. After assigning co-expression scores for each sample, we found differentially co-expressed interacting protein pairs (DEPs) among conditions for a condition-specific signature. We applied the DEP-based method to samples representing the clinical stages of HIV-1 infection to discover an HIV-1 stage-specific signature.

## Methods

### Acquisition of HIV-1-infected gene expressions and human protein–protein interactions

**Figure 1:**
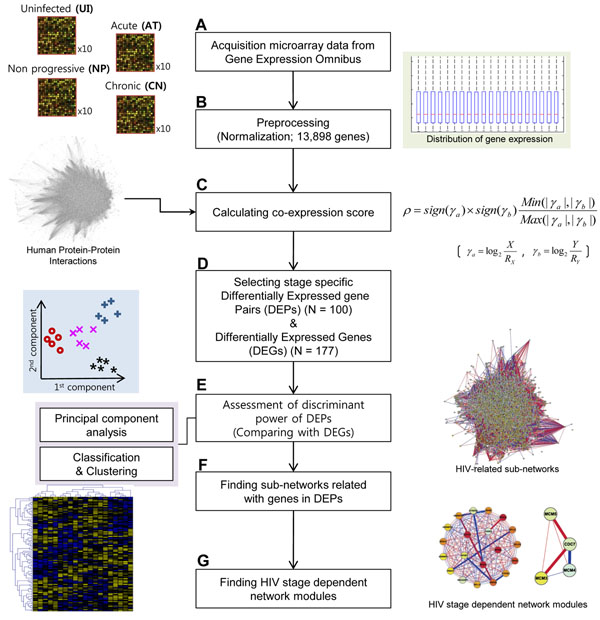
**Study overview.** After acquiring expression data from gene expression omnibus (**A**, **B**), differentially expressed genes (DEGs) and differentially expressed gene pairs (DEPs) were selected and evaluated for usefulness (**C**–**E**). The results of network analysis determined the HIV-related network modules (**F**, **G**).

For HIV-1 expression data, we downloaded the Series GSE6740 dataset from the GEO database (http://www.ncbi.nlm.nih.gov/geo). The dataset contains stage-specific gene expressions of CD4+ and CD8+ cells from a cohort of HIV-infected individuals [5]. The HIV-infected individuals had not been treated at the time samples were obtained. The profiles of CD4+ and CD8+ T cells from individuals with early HIV-1 infections (*Acute*), non-progressive HIV-1 infections with low or undetectable viral loads (*Non-progressive*), chronic progressive HIV-1 infections (*Chronic*), and uninfected individuals (*Uninfected*) were selected (**Figure**1A). The expression profiles were normalized using a quantile normalization method implemented in MATLAB R2009b (Mathworks, Natick, MA, USA) (**Figure**1B). Final expression datasets contained 10 *Acute*, 10 *Non-progressive*, 10 *Chronic* samples, and 10 *Uninfected* samples. For human protein–protein interactions (PPIs), we used the data of Lee *et al. *[10], which incorporated public databases such as DIP [11], BIND [12], HPRD [13], and REACTOME [14]. The data set also included the results of several recent genome-wide studies [15-18]. A total of 80 970 interactions among 10 819 human proteins were prepared. For the subsequent analyses, including calculating co-expression scores and identifying differentially co-expressed interacting protein pairs, only these interactions were considered.

### Calculating a co-expression score between two interacting gene products

A co-expression (or co-changing) score between two interacting gene products in a sample was calculated by following two steps (**Figure**1C). First, expression-changing ratios (*γ*) of two genes, *a* and *b*, were defined as follows:

where *X* (or *Y*) indicates the normalized expression level of gene *a* (or *b*), and *R_X_* (or *R_Y_*) is a representative expression level of gene *a* (or *b*). In this study, the median value of expressions across samples was used as a representative value of a specific gene. Next, a co-expression score (*ρ*) between the two interacting gene products in the sample was calculated using the following equation:

where *Min* (*p*, *q*) or *Max* (*p*, *q*) indicates the minimum or maximum value between *p* and *q*, respectively, and *sign* (*x*) indicates the sign of *x*. Note that the co-expression score *ρ* has a positive value if *X* and *Y* are simultaneously greater (or smaller) than *R_X_* and *R_Y_*; otherwise, it has a negative value. Moreover, the co-expression score *ρ* has a higher absolute value if the absolute values of expression-changing ratios are similar between the two genes.

### Identifying DEPs and DEGs

To identify DEPs for HIV-1 infection, analysis of variance (ANOVA) and geometric means of differences between median co-expression scores across individual stages were used (**Figure**1D). An interaction with a high value of -log_10_(p-value of an ANOVA test) × (geometric mean of differences of median co-expression scores) was considered significant, and interactions with higher degrees of significance than a specific cutoff value were selected as DEPs. A similar process was applied to select DEGs, except that expression levels, not co-expression scores, were used. To ensure a balanced comparison, the most highly significant DEGs were selected in a quantity equal to the number of genes. Here, several values from 0.5 to 1 were examined to identify the optimal cutoff value that provided the smallest number of DEPs and DEGs with the best accuracy.

### PCA, clustering, and classification analyses of DEPs and DEGs

To estimate how DEPs reveal HIV-1 stage-specific characteristics, principal-component analysis (PCA) and several well-known classification and clustering methods were used (**Figure**1E). PCA transforms attribute values into new ones to create the linear projection of the data that accounts for the most variance in a low-dimensional subspace. Therefore, it provides snapshots of data that we can see at a glance. Global views of DEPs were compared with those of whole genes and DEGs using PCA. PCA was performed using the algorithm implemented in MATLAB, R2009b (MathWorks, Natick, MA, USA).

For clustering, we used hierarchical clustering (HCL) with the K-means methods implemented in MEV4.0 (Multiple Experiment Viewer, http://www.tm4.org) [19]. The HCL method groups samples according to the degree of similarity between them based on feature information (here, DEPs or DEGs) without considering the class information (here, the HIV-1 stages). Therefore, it was possible to confirm whether the selected feature information of samples (i.e., DEPs or DEGs) is valuable for clustering samples according to stage. K-means clustering, like HCL, is an unsupervised learning method. However, K-means clustering was used to partition DEPs (or DEGs) into some number of clusters. Here, DEPs were clustered into six groups in each cell type (CD4+ and CD8+ cells). Stage-specific clusters in both CD4+ and CD8+ cells were then further characterized through GO term analysis.

For classification methods, we used the J48 decision tree, the SMO support-vector machine, and the multilayer perception artificial neural network, which were implemented in WEKA, version 3.6.3 [20]. Leave-one-out cross-validation (LOOCV) using these methods was applied to estimate the classification performance of selected DEPs (or DEGs) for predicting the disease stages of HIV-1. LOOCV is one of the most popular validation methods; it trains prediction models using all samples except one and then tests the models with the remaining sample. This step then passes through as many iterations as there are samples [21]. For performance measures, we used accuracy, sensitivity, and specificity from a confusion matrix.

### DEP-based network-module identification

To discover HIV-related interaction sub-networks, the prepared PPIs and the list of genes in DEPs were imported into Cytoscape (http://www.Cytoscape.org) [22] with the median co-expression score for each stage (**Figure**1F). Next, we included the genes that directly interacted with the genes in DEPs. Using the extended network, MCODE was used to find sets of genes located at the area of dense DEPs (**Figure**1G). MCODE is a Cytoscape plug-in and is one of the most popular methods by which to find highly interconnected regions in a network [23]. The score of a sub-network was calculated based on the complexity and density of the network. The top 10 modules with the highest network scores were considered significant since modules with higher network scores showed higher prediction accuracies in previous study [23]. Among 10 significant modules, five modules containing DEPs were finally selected because none of the other five modules included any DEP. Note that a DEP in each sample has its own co-expression score. To find a representative HIV-related module under a specific stage, thus, we used a median value of multiple co-expression scores for an interaction and a median expression level for a protein, respectively.

### Gene-ontology enrichment analysis

For the gene list in DEPs and DEGs, a functional annotation tool called the Database for Annotation, Visualization and Integrated Discovery (DAVID) [24] was applied to find functionally enriched terms. DAVID uses a Fisher’s exact test to determine whether the proportion of selected genes falling into each category differs from the baseline (here, all genes of *Homo sapiens*). For selected modules, BiNGO (the Biological Networks Gene-ontology tool) [25] was also used to conduct gene-ontology (GO) enrichment analysis. BiNGO, which is implemented as a plug-in for Cytoscape, maps the predominant functional themes of a given gene set on the GO hierarchy and outputs this mapping as a Cytoscape graph. Hypergeometric distribution was adopted to find a functional degree of overrepresentation of an HIV-related module using this method.

## Results

### Identifying DEPs across HIV-1 stages

**Figure 2:**
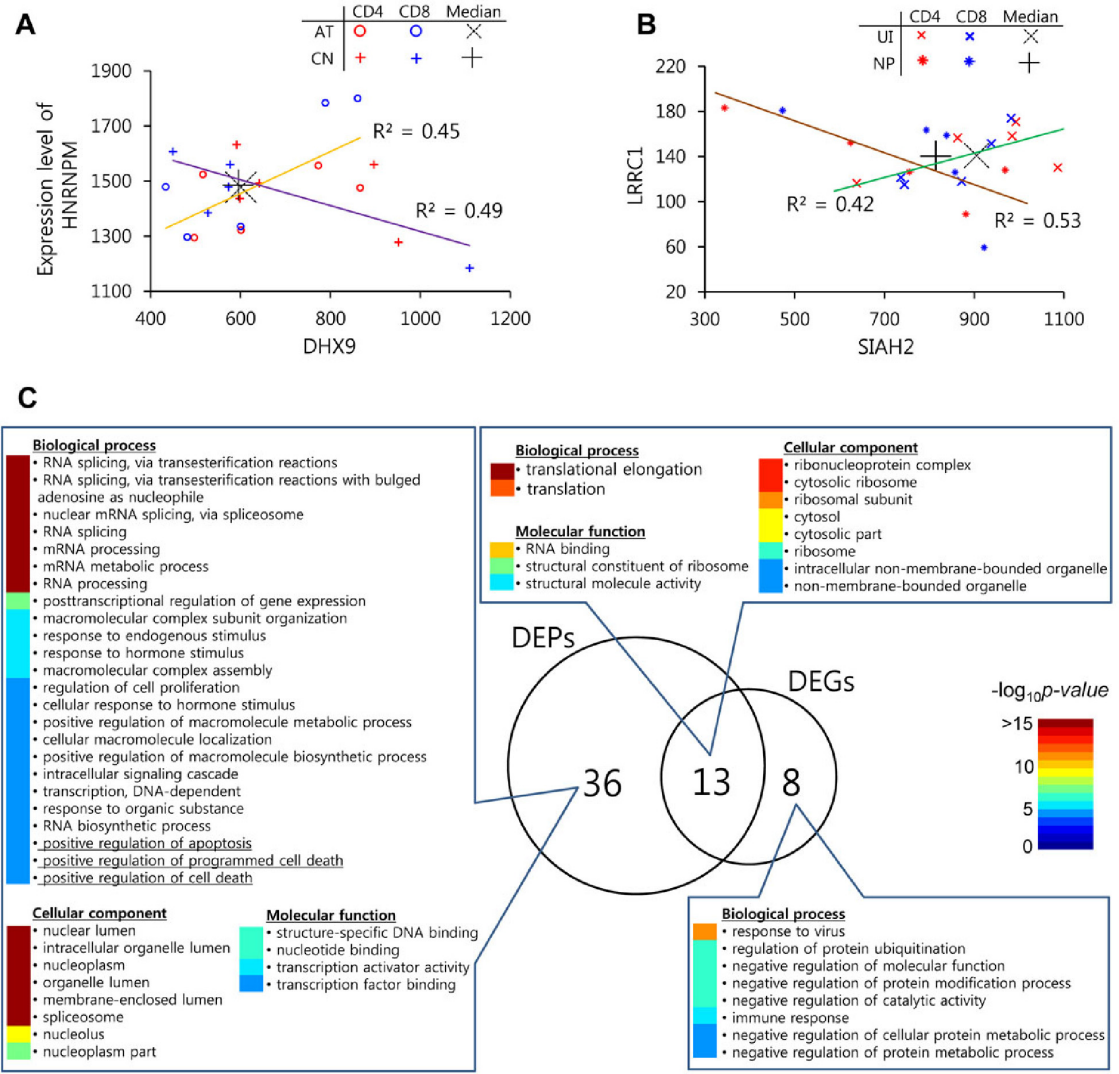
**Comparison of DEPs and DEGs.** (**A**, **B**) Expressions and correlations of DEPs. The *X*- and *Y*-axes represent expression levels of each gene. Expression levels of the samples are represented by dots (X, *Uninfected* (UI); O, *Acute* (AT); *, *Non-progressive* (NP); +, *Chronic* (CN)). Median values of samples in the same stage are marked with bigger black “+” and “×”. Trend lines are shown with degrees of correlation. (**C**) Gene-ontology (GO) enrichment analysis of DEPs and DEGs. The numbers in circles indicate the counts of GO terms relevant to DEPs, DEGs, or both. Precise details and their -log_10 _*p* values are listed next to the numbers.

We prepared 10 *Acute*, 10 *Non-progressive*, 10 *Chronic* samples, and 10 *Uninfected* samples of CD4+ and CD8+ cells from a cohort of HIV-infected individuals (**Methods**). For human protein-protein interactions, we used the data of Lee *et al. *[10], which cover 80 970 interactions among 10 819 proteins. To identify DEPs, we first calculated a co-expression score of each interaction using expression-changing ratios of the corresponding two proteins. After that, we found significantly different pairs using analysis of variance (ANOVA) and geometric means of differences of median levels across HIV-1 stages. Among 80 970 interacting protein pairs, 100 pairs were selected as significant DEPs which consist of 177 proteins across HIV-1 infection (**Table**1). A DEG-based analysis, on the other hand, selected a different set of 177 genes: only eight genes (or their corresponding encoding proteins) were common to DEPs and DEGs. **Figures**2A and 2B show some cases of the selected DEPs in which DEPs could differentiate between pathophysiologically similar HIV-1 stages. For example, the co-expression pattern, illustrated by the line in the figure, between HNRNPM (heterogeneous nuclear ribonucleoprotein M, known to influence pre-mRNA processing and other aspects of mRNA metabolism and transport) and DHX9 (DEAH (Asp-Glu-Ala-His) box polypeptide 9, known to be involved in the expression and nuclear export of retroviral RNAs and also known to interact with HIV-1 RNA) was positively related to the *Acute* stage, whereas it was negatively related to *Chronic* stage (**Figure**2A). However, a DEG-based analysis missed the HIV-1 related proteins HNRNPM and DHX9 because of the similar median expression levels across the *Acute* (black “+”) and *Chronic* (black dashed “×”) stages. A similar phenomenon was observed between LRRC1 (leucine-rich repeat containing 1) and SIAH2 (seven in absentia homolog 2, known to be involved in ubiquitination and proteasome-mediated degradation) which were positive in *Uninfected* but negative in *Non-progressive* stages, whereas the median expression levels of LRRC1 and SIAH2 were similar between the two stages (**Figure**2B).

**Table 1:** Protein pairs included in the top-30 DEPs

Protein 1	Name of Protein 1	Protein 2	Name of Protein 2	P-value	G-mean	Significance
NUTF2	nuclear transport factor 2	NUP62	nucleoporin 62kDa	7.5E-06	0.37	1.90
CDC7	cell division cycle 7 homolog (S. cerevisiae)	MCM3	minichromosome maintenance complex component 3	1.3E-05	0.34	1.66
VAMP1	vesicle-associated membrane protein 1 (synaptobrevin 1)	ARFGAP1	ADP-ribosylation factor GTPase activating protein 1	4.3E-05	0.36	1.56
HSPA8	heat shock 70kDa protein 8	TADA3L	transcriptional adaptor 3	1.0E-05	0.26	1.29
TNR	tenascin R (restrictin, janusin)	NFASC	neurofascin	1.6E-04	0.33	1.25
ARHGEF2	Rho/Rac guanine nucleotide exchange factor (GEF) 2	PRKCI	protein kinase C, iota	6.4E-05	0.28	1.17
PDGFRB	platelet-derived growth factor receptor, beta polypeptide	SNX2	sorting nexin 2	8.7E-05	0.28	1.14
RFC5	replication factor C (activator 1) 5, 36.5kDa	POLA1	polymerase (DNA directed), alpha 1, catalytic subunit	5.5E-05	0.27	1.13
NFIB	nuclear factor I/B	RFX1	regulatory factor X, 1 (influences HLA class II expression)	3.4E-04	0.32	1.12
EIF3I	eukaryotic translation initiation factor 3, subunit I	SUMO4	SMT3 suppressor of mif two 3 homolog 4 (S. cerevisiae)	1.7E-05	0.23	1.09
COL17A1	collagen, type XVII, alpha 1	LAD1	ladinin 1	3.9E-04	0.31	1.07
IRS1	insulin receptor substrate 1	UBTF	upstream binding transcription factor, RNA polymerase I	4.5E-04	0.32	1.05
CAV1	caveolin 1, caveolae protein, 22kDa	TRAF6	TNF receptor-associated factor 6	1.0E-04	0.26	1.05
RPS14	ribosomal protein S14	RPS27A	ribosomal protein S27a	3.2E-03	0.41	1.03
HNRNPA2B1	heterogeneous nuclear ribonucleoprotein A2/B1	HNRNPH1	heterogeneous nuclear ribonucleoprotein H1 (H)	4.1E-05	0.23	1.02
TAF4	TATA box binding protein (TBP)-associated factor, 135kDa	CBX3	chromobox homolog 3	1.0E-03	0.34	1.01
VPS11	vacuolar protein sorting 11 homolog (S. cerevisiae)	VPS45	vacuolar protein sorting 45 homolog (S. cerevisiae)	2.4E-04	0.28	1.00
ATP5F1	ATP synthase, H+ transporting, mitochondrial Fo complex, subunit B1	ATP5J2	ATP synthase, H+ transporting, mitochondrial Fo complex, subunit F2	8.1E-05	0.24	0.99
POLR2G	polymerase (RNA) II (DNA directed) polypeptide G	SF3B2	splicing factor 3b, subunit 2, 145kDa	2.6E-03	0.38	0.98
PDPK1	3-phosphoinositide dependent protein kinase-1	PRKCQ	protein kinase C, theta	4.5E-05	0.22	0.97
EP300	E1A binding protein p300	TF	transferrin	5.3E-04	0.30	0.97
RPS5	ribosomal protein S5	RPL28	ribosomal protein L28	1.4E-03	0.34	0.96
ELK1	ELK1, member of ETS oncogene family	GRB10	growth factor receptor-bound protein 10	9.7E-04	0.32	0.96
RPS13	ribosomal protein S13	ATAD3A	ATPase family, AAA domain containing 3A	5.8E-05	0.23	0.95
PABPC1	poly(A) binding protein, cytoplasmic 1	RPS4Y1	ribosomal protein S4, Y-linked 1	2.5E-03	0.37	0.95
PRPF4	PRP4 pre-mRNA processing factor 4 homolog (yeast)	PPIH	peptidylprolyl isomerase H (cyclophilin H)	6.5E-04	0.29	0.93
ZFP36	zinc finger protein 36, C3H type, homolog (mouse)	EIF2C4	eukaryotic translation initiation factor 2C, 4	6.3E-04	0.29	0.93
RPLP2	ribosomal protein, large, P2	RPL29	ribosomal protein L29	1.2E-03	0.32	0.93
HSF1	heat shock transcription factor 1	STIP1	stress-induced-phosphoprotein 1	1.5E-04	0.24	0.92
GSK3B	glycogen synthase kinase 3 beta	FUS	fused in sarcoma	2.7E-03	0.35	0.91

Here, significance is “-log(P-value) × G-mean”.

The functional characteristics were also significantly different between DEPs and DEGs (**Figure**2C). The number of enriched GO terms using the 177 proteins of DEPs was 49, whereas it was 21 in the case of DEGs (the GO terms with >10 genes/proteins and *p*-value of <1.0×10^–5^ using DAVID tools). Among the enriched GO terms, 13 overlapped and were mainly associated with “translation” biological processes. Thirty-six GO terms included only in DEPs were related to responses against endogenous or exogenous stimuli (from transcription to mRNA processing). They were particularly associated with apoptosis (“positive regulation of apoptosis”, “positive regulation of programmed cell death”, and “positive regulation of cell death”), which is known to be an important factor in the progression of HIV by the resulting depletion of T helper cells [26]. On the other hand, the GO terms only for DEGs included “response to virus” and “immune response”.

### PCA results of DEPs and DEGs of HIV-1

**Figure 3:**
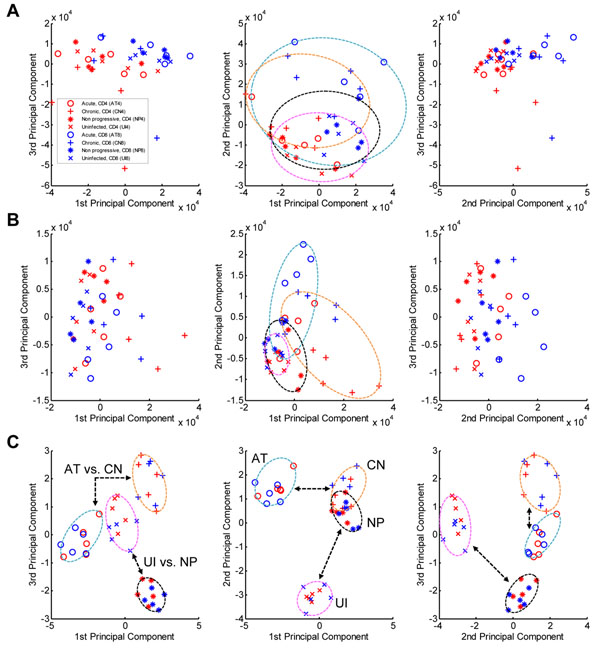
**Result of principal-component analysis.** (**A**) Global view of all expression data sets using principal component analysis. The first principal component accounts for as much variability in the expression of total genes as possible. The second and third components account for as much of the remaining variability as possible. Results of principal component analysis with DEGs and DEPs are shown in (**B**) and (**C**), respectively.

We applied PCA to evaluate the geometric view of the samples in various HIV-1 stages with *i*) whole genes of the microarray, *ii*) the identified DEGs, or *iii*) the identified DEPs (**Figure**3). In the case of whole genes (**Figure**3A), the four areas of samples in distinct HIV-1 stages highly overlapped one another with regard to the first three principal components, even though the samples were separable according to cell type (CD4+ cells in red and CD8+ cells in blue). In the PCA analysis with DEGs (**Figure**3B), the HIV-1 stages were still not separable; in particular, the *Uninfected* and *Non-progressive* areas and the *Acute* and *Chronic* areas were highly overlapped. When the identified DEPs were used (**Figure**3C), however, all four stages were clearly separable using the three components, regardless of cell type. Interestingly, pathophysiologically similar HIV-1 stages, such as *Acute* and *Chronic* or *Uninfected* and *Non-progressive*, were highly discriminable using the first two principal components (middle panel of **Figure**3C), although there was an area that overlapped between *Chronic* and *Non-progressive*. These four stages were clearly separable both in the first and third planes and in the second and third planes (first and third panels of **Figure**3C, respectively). This tendency was also observed in individual cell types, i.e., in CD4+ cells and CD8+ cells. The DEP PCA results showed the most clearly discriminated distribution of the samples across the HIV-1 stages compared with those of whole genes or DEGs regardless of cell type.

### Clustering results of DEPs in HIV-1

**Figure 4:**
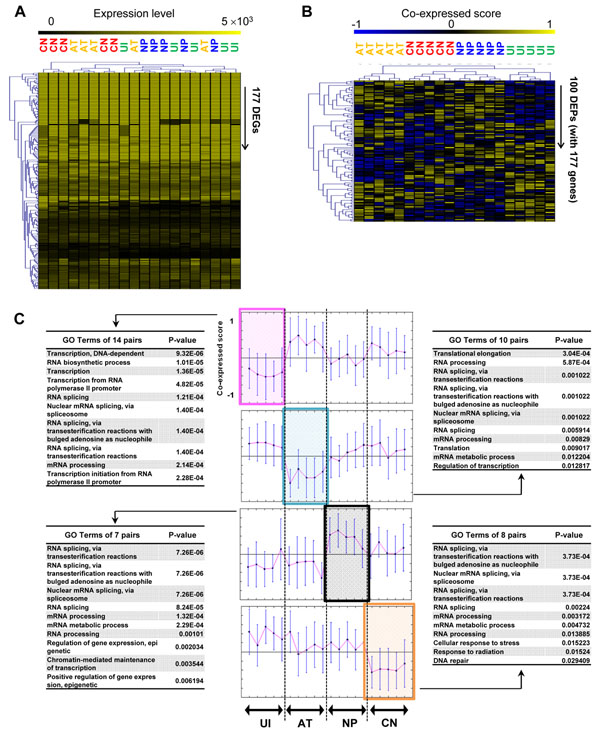
**Clustering results using DEPs and DEGs.** (**A**) Heat map for expression of DEGs across the samples. Each lane represents the expression profile of one sample. Result of hierarchical clustering with DEGs is shown at the top of the heat map (UI, *Uninfected*; AT, *Acute*; NP, *Non-progressive*; CN, *Chronic*). (**B**) Heat map for co-expressed score of DEPs. (**C**) Four representative clusters of gene pairs. Of six groups of gene pairs clustered by K-means clustering using Pearson’s correlation between pairs and samples, four groups had relatively different co-expression scores compared with the other stages. The *X*-axis represents samples, and the *Y*-axis represents co-expression score. The top 10 GO terms related to biological processes are listed in descending order of *p*-values.

We applied clustering methods to the samples without seeing their HIV-1 stage information. When the extracted DEG-based features were used for the individual CD4+ samples (**Figure**4A), the samples were mixed together across the different HIV-1 stages, especially between *Acute* and *Chronic* (in orange and red, respectively) and between *Non-progressive* and *Uninfected* (in blue and green, respectively). This indicates that *Chronic* samples and *Acute* samples have similar expression patterns, as do *Non-progressive* and *Uninfected* stages. When DEP-based features were used with the same hierarchical clustering method, however, same-stage samples were clustered first (**Figure**4B). After grouping samples according to stage information, *Chronic* and *Non-progressive* samples were then clustered. Finally, *Uninfected* and *Acute* were clustered in sequence. The heat maps of hierarchical clustering results showed that the co-expression patterns of each stage were quite different across the HIV-1 stages, whereas individual gene expression patterns lacked distinct patterns according to stage, especially between pathophysiologically similar HIV-1 stages (*e.g.*, between *Acute* and *Chronic* or between *Uninfected* and *Non-progressive* stages). This tendency was also observed in CD8+ cells. Moreover, the DEP-based features clearly clustered each stage of HIV-1, regardless of cell type. Co-expression patterns of samples were preserved across individual HIV-1 stages.

To discover stage-specific co-expressed pairs, we next clustered the 100 identified DEPs into six groups using a K-means clustering method. Different cell types might be associated with different DEP groups; therefore, we identified separate clusters using only CD4+ samples or only CD8+ samples. Of the six groups, four groups in each cell type showed stage-specific co-expression patterns. Surprisingly, the median co-expression scores of groups across samples were quite similar between CD4+ and CD8+ cells. Moreover, each pair of groups with a similar co-expression pattern shared many DEPs. Using the DEPs shared between CD4+ and CD8+ cells, we analyzed enriched GO terms (**Figure**4C). Common biological functions of all four clusters were “RNA splicing” and “mRNA processing”. The first cluster, which was composed of 14 DEPs, had lower co-expression scores only in the *Uninfected* stage. The genes of the 14 DEPs are known to play a major role in “transcription” in addition to the common functions. In contrast, the second cluster, which had 10 DEPs with lower co-expression scores only in the *Acute* stage, was involved in “translation” rather than “transcription”. The genes in the seven DEPs of the third cluster, which had higher co-expression scores only in the *Non-progressive* stages, are also known to play a role in “epigenetic processing”. Major functions of the last cluster (*Chronic*) were related to “repair processes” such as “cellular response to stress”, “response to radiation” and “DNA repair”.

### Discriminant power of DEPs for HIV-1 stages

**Figure 5:**
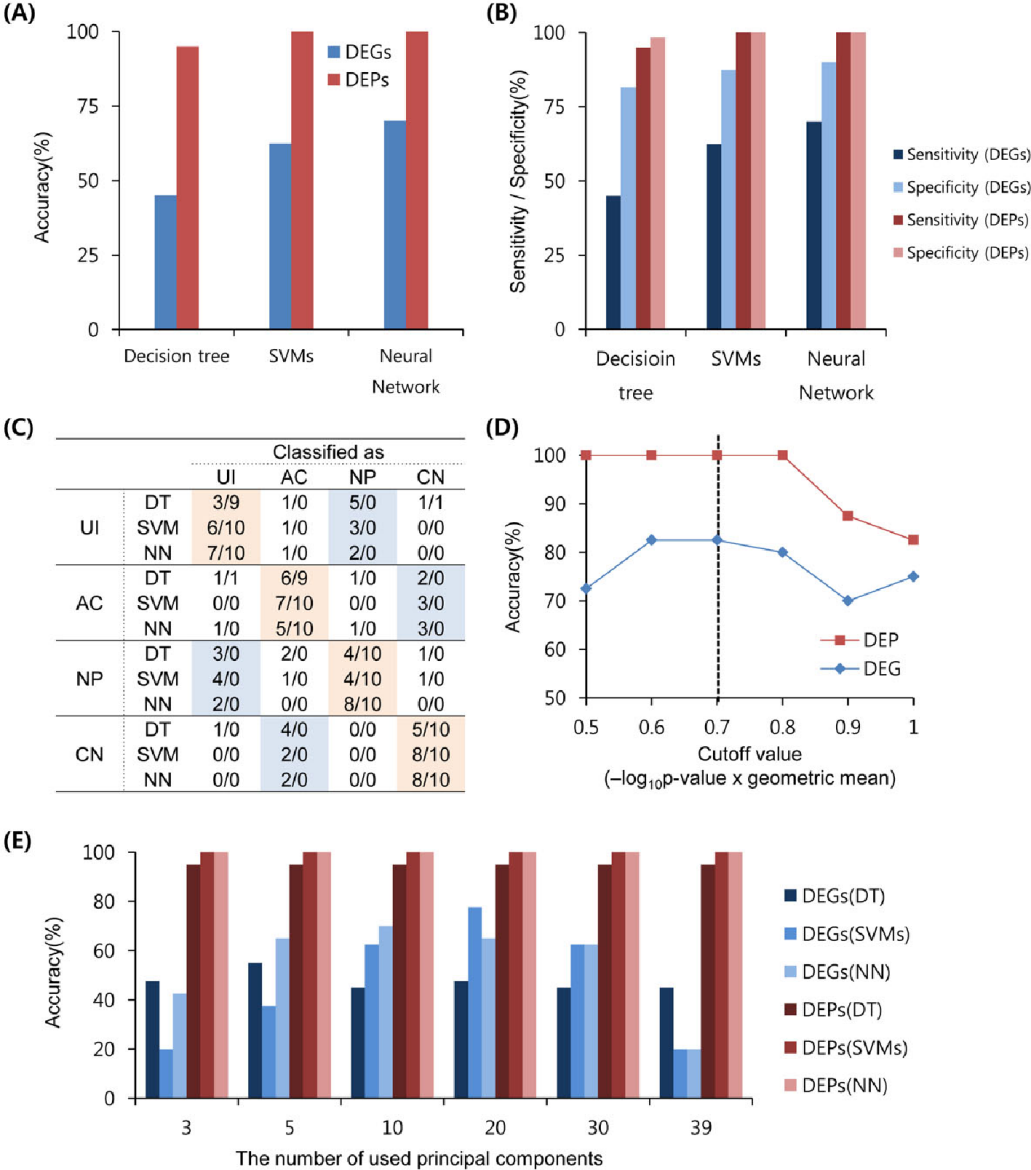
**Performance of classification models using DEPs and DEGs.** (**A**) Accuracies of the DEP- and DEG-based models. The accuracy of all models was estimated by the leave-one-out test. (**B**) Sensitivities and specificities of the DEP- and DEG-based models. (**C**) Confusion matrix of DEP- and DEG-based models in (A) (UI, *Uninfected*; AT, *Acute*; NP, *Non-progressive*; CN, *Chronic*; DT, Decision tree; NN, Neural network). This matrix shows the actual stages of samples and their predicted stages by classification methods. Each column represents a predicted stage, and each row represents an actual stage. The count represents the result for DEGs/that for DEPs. (**D**) Accuracy of SVMs according to cutoff values for selecting DEPs. (**E**) Accuracies according to classification models and the number of principal components used for building models. DEPs showed higher accuracy than did DEGs regardless of the classification model or the number of used principal components.

To directly investigate the discriminant power of DEPs for the HIV-1 stages, we compared the prediction performance of the identified DEPs with that of the DEGs using several well-known classification methods including a decision tree, a support vector machine, and an artificial neural network (**Methods**). We here used a LOOCV approach with several performance measures including accuracy, sensitivity, and specificity. As shown in **Figure**5A, the prediction accuracies (95%, 100%, and 100%, respectively) of DEPs were much higher than those (45%, 62.5%, and 70%, respectively) of DEGs regardless of the classification method used. The better performance of DEPs was also observed with other measures including sensitivity and specificity (**Figure**5B). Moreover, among the misclassification cases, the 71.4% incorrect prediction (35 among 49 wrong predictions) of the three models built with DEGs was caused by misclassification between *Acute* and *Chronic* or between *Uninfected* and *Non-progressive* (**Figure**5C). In the case of DEPs, however, the three models did not misclassify any case between these stages. The only two misclassified cases were misclassification of an *Uninfected* sample as *Chronic* and an *Acute* sample as *Uninfected*. The DEP-based features correctly classified the pathophysiologically similar HIV-1 stage pairs.

Next, we evaluated the influence of the number of DEPs (or DEGs) on the power for discriminating the HIV-1 stages. The performance was evaluated by a support-vector machine with various cutoff values for the degree of significance of DEPs from 0.5 to 1.0. Here, as many DEGs were selected as there were DEP genes. As shown in **Figure**5D, the highest accuracy was achieved at values of 0.6 and 0.7 (100% accuracy in classifying HIV-1 stages with DEPs and 82.5% accuracy with DEGs). Thus, in this study, we selected 0.7 as the cutoff value for DEP selection because it revealed the highest level of performance with a smaller number of DEPs and DEGs. As a result, 100 DEPs consisting of 177 genes and 177 DEGs were selected. Additionally, we investigated the impact of the number of selected features from PCA analysis with the selected DEPs and DEGs. In the PCA analysis using the 177 DEGs, about 20 principal components were required to achieve the best performance (**Figure**5E). For the selected DEP cases, however, only three principal components were required to obtain the best performance, which was the same accuracy as given by all components of PCA using DEPs. This also suggests that DEP-based features have meaningful discriminant information with respect to the HIV-1 stages.

### Discovery of HIV-1 stage-specific network modules using DEPs

**Figure 6:**
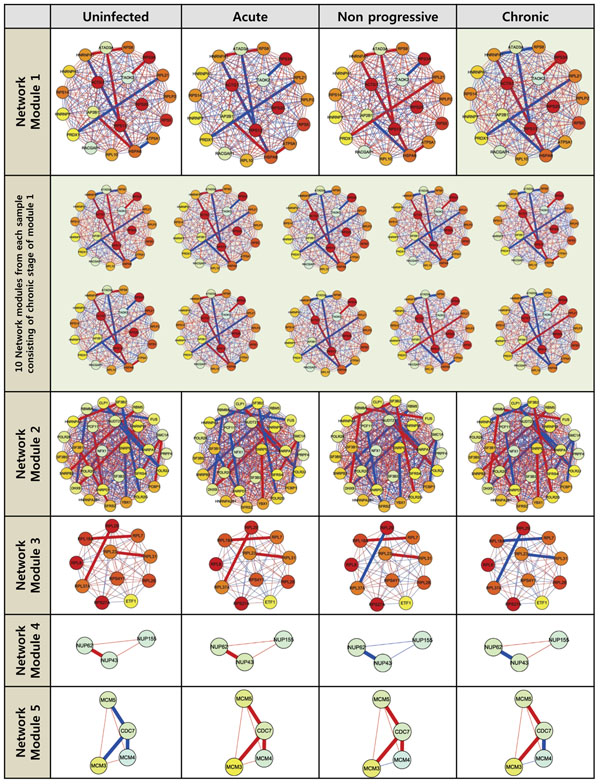
**HIV-related network modules.** Thin lines represent PPI, and thick lines denote DEPs. Nodes indicate protein, and node color denotes expression level. Blue, yellow, and red represent increasingly higher expressions of genes. The color of the edge represents the sign of the co-expressed score. If the sign is positive, the edge color is red, and if it is negative, blue is used. Representative modules for each stage were created using the median value of the co-expressed score and expression level of samples in the same stage. Network module 1 of the *Chronic* stage was made from 10 network modules listed in the second row.

From the selected DEPs, we discovered HIV-1 stage-specific network modules. In this analysis, we also included genes that directly interact with the genes in the DEPs to extend the genes in DEPs. The extended network had 3 545 nodes representing genes, with 50 739 edges between nodes denoting PPIs including DEPs. Using the extended network and an MCODE method (**Methods**), we identified five HIV-1-related network modules across the HIV-1 stages (**Figure**6). Their network scores were 51.8, 45.0, 28.5, 14.0, and 11.0, respectively. Genes and interactions that composed a network module were predefined in the module-searching phase, but each model of a specific HIV-1 infection stage had its own representative co-expression scores and expression levels. In network module 5, for example, the co-expression score between CDC7 and MCM3 was negative only in the *Uninfected* stage. Additionally, the interactions between CDC7 and MCM4 had identical signs for the co-expression score in *Uninfected* and *Chronic* stages or in *Acute* and *Non-progressive* stages, respectively. In GO enrichment analysis (**Methods**), all identified network modules were related to DNA or RNA metabolisms. More specifically, the main functional categories of the biological process of network modules 1 and 3 were “translation,” including “translational elongation,” “translation,” and “gene expression.” Module 2 was related to “RNA splicing,” including “nuclear mRNA splicing,” “RNA splicing via transesterification reactions,” and “mRNA processing.” Module 4 was related to “mRNA, RNA, and nucleic acid transport,” and module 5 was associated with “DNA metabolism,” including “DNA replication” and “DNA metabolic process.” The HIV-related network modules revealed by network analysis using DEPs correspond with the results of some earlier studies (see **Discussion** for detailed explanation). The proposed DEP-based method, therefore, complemented the DEG-based approach in the microarray expression analysis of HIV-1 infection.

## Discussion

The purpose of this study was to develop a novel microarray data analysis method to discover the stage-specific protein pairs in HIV-1 infection. The developed novel method focuses on the expression co-changing patterns between interacting protein pairs rather than on expression levels of individual genes. Note that we here only considered known PPIs that contact physically or chemically in selecting DEPs rather than all possible pairs among all detected genes in microarray; both because expressions noisy and because physically or chemically contacting pairs can share biological function and thus their biological meaning can be easily interpreted. Strength of our method comparing existing correlation-based method is that it can capture a genetic signature for a single sample. Even though one or more samples are used for selecting representative expression level, our method can identify a genetic signature for a new single sample by comparing with known representative expression level if those levels were already known by previous study.

With this method, 100 DEPs were selected for the discriminant features of HIV-1 stages. A comparison between DEPs and DEGs revealed that DEPs more powerfully classified the ambiguous stages of HIV-1. This means that DEPs can provide additional information not included in DEGs. As shown in **Figure**2A, for example, the HIV-1-related proteins HNRNPM (600.8 under *Acute* and 595.0 under *Chronic*) and DHX9 (1477.7 under *Acute* and 1485.7 under *Chronic*) had similar expression levels between *Acute* and *Chronic* stages even though the variations within individual stages were relatively large (*i.e.*, the expression level of DHX9 ranged from 420 to 1110 in the *Chronic* stage). Thus, the previous DEG-based approach missed both HNRNPM and DHX9 as the stage-specific genes for HIV-1. However, if we consider an expression co-changing pattern, the HNRNPM and DHX9 pair selected a significant feature of the HIV-1 stages because the co-changing scores were consistently positive in *Acute* samples but consistently negative in *Chronic* samples. In this respect, the DEP-based approach could well discriminate all four stages of HIV-1. Moreover, DEPs were enriched in more HIV-related GO terms, such as “apoptosis”, which is strongly associated with the spectrum of the progression of HIV infection. Additionally, there is distinct difference between the DEP-based approach and previous correlation-based network analyses [7,8]. The biggest difference is that the DEP-based approach generates a distinct feature set with only one sample, whereas a correlation-based network approach finds a network feature with groups of samples under a specific condition. Thus, it is difficult to capture the characteristics of individual samples using the previous correlation-based network analyses. If there is a problem in predicting an unknown or new sample and if DEG-based analysis is unclear, then the DEP-based approach might be applicable.

The HIV-related network modules revealed by network analysis using DEPs correspond with the results of some earlier studies. Heterogeneous nuclear ribonucleoproteins (hnRNPs; complexes of RNA and protein) in modules 1 and 2 are known as HIV protein-synthesis modulators [27]. In module 2, SF3B2 (splicing factor 3b, subunit 2, 145 kDa) modulates viral proliferation of HIV through interaction with Vpr (Viral Protein R) [28]. SFRS2 (serine/arginine-rich splicing factor 2) influences the use of the HIV-1 splicing site [29]. SNRPE (small nuclear ribonucleoprotein polypeptide E), one of the transcription elongation complexes, assembles with HIV Tat [30]. DHX9 (DEAH (Asp-Glu-Ala-His) box polypeptide 9) affects the expression of HIV-1 [31]. Furthermore, there is an association between PCF11 (PCF11, cleavage and polyadenylation factor subunit, homolog) and HIV-1 transcription[32]. PCBP1 (poly(rC) binding protein 1) and YBX1 (Y-box binding protein 1) interact with Rev protein, a key regulator of HIV-1 gene expression [33]. NUP62 (nucleoporin 62 kDa) is related to Rev-mediated viral RNA export by interacting with eIF-5A (eukaryotic translation-initiation factor 5A) [34]. In contrast to NUP62, NUP155 (nucleoporin 155 kDa) is associated with the import of HIV DNA [35]. All of these genes were included in DEPs but not in DEGs. From the module analysis with DEPs, it seems that changes in DNA and RNA metabolism are crucial in the clinical manifestations of HIV infection, and DEPs and HIV-related network modules might have the potential to assist in the elucidation of the pathogenesis of HIV-1 infection at the genomic and proteomic levels. However, further studies to seek biological confirmation are imperative to clarify the detailed roles of DEPs in specific HIV-1 stages.

## Conclusions

We present a novel microarray data analysis method based on DEP by focusing on the expression co-changing patterns between interaction pairs. The DEP based algorithm was more powerful in classifying the ambiguous stages of HIV-1 and revealed the HIV-1 stage-specific network modules. The DEP-based method might contribute to complementation of existing DEG-based analyses.

## Competing interests

The authors have no conflicts of interest to declare.

## Authors' contributions

Yoon, Lee contributed to the study conception and design. Yoon, Kim and Lee contributed to acquisition of data, analysis and interpretation of data. Yoon, Kim and Lee were responsible for drafting the manuscript. All authors have involved in revising the manuscript and have given final approval of the version to be published.
